# Estimation of disability free life expectancy in non small cell lung cancer based on real world data

**DOI:** 10.1038/s41598-023-40117-5

**Published:** 2023-08-16

**Authors:** Shin-Mao Lin, Szu-Chun Yang, Tzu-I. Wu, Jung-Der Wang, Li-Fan Liu

**Affiliations:** 1 Department of Environmental and Occupational Medicine, National Cheng Kung University Hospital, College of Medicine, Tainan, Taiwan; 2 Department of Internal Medicine, National Cheng Kung University Hospital, College of Medicine, National Cheng Kung University, Tainan, Taiwan; 3 Department of Gerontology, College of Medicine, National Cheng Kung University, Tainan, Taiwan; 4 Department of Environmental and Occupational Medicine, National Cheng Kung University Hospital, College of Medicine, National Cheng Kung University, Tainan, Taiwan; 5 Department of Geriatric, National Cheng Kung University Hospital, College of Medicine, National Cheng Kung University, No.138, Sheng-Li Road, Tainan, 70428 Taiwan

**Keywords:** Geriatrics, Public health, Quality of life

## Abstract

To quantify the societal impact of disability in patients with non-small cell lung cancer (NSCLC), this study estimated the disability-free life expectancy (DFLE), loss-of-DFLE and explored their associations with quality-adjusted life expectancy (QALE) and loss-of-QALE. We interlinked national databases and applied a rolling-over algorithm to estimate the lifetime survival function for patients with NSCLC. Using the EuroQOL-5 Dimension (EQ-5D) and Barthel index (BI), we repeatedly measured the quality-of-life and disability functions of NSCLC patients who visited our hospital from 2011 to 2020. Age-, sex-matched referents were simulated from lifetables of the same calendar year of diagnosis. We categorized BI scores ≤ 70 as in need of long-term care and constructed linear mixed models to estimate the utility values and disability scores. We collected 960 cases and 3088 measurements. The proportions of measurements without disability at age 50–64 and in stage I–IIIa, 50–64 and stage IIIb–IV, 65–89 and stage I–IIIa and 65–89 and stage IIIb–IV were 97.3%, 89.3%, 94.8%,78.3%, corresponding to DFLEs of 15.3, 2.4, 6.8, 1.2 years and losses-of-DFLE of 8.1, 20.7, 4.0, 8.6 years, respectively, indicating that advanced stage had a stronger effect than old age. Survivors in advanced stages showed increased demands for assistance in almost all subitems. The DFLEs seemed to be approximate to the QALEs and the latter were shorter than the former due to discomfort and depression. From a societal perspective, future health technology assessment should consider the impact of lifetime duration of functional disability. Early diagnosis of NSCLC may decrease the burden of long-term care.

## Introduction

Cancer has been one of the top leading causes of death worldwide for the last several decades and 10 million deaths were attributed to cancer in 2020^1^.

According to the statistical data provided by the Taiwan Ministry of Health and Welfare, cancer has been ranked as the leading cause of mortality in Taiwan for more than three decades^2^. Among various types of cancer in Taiwan, lung cancer exhibited the second-highest incidence rate and the highest mortality rate^2,3^.

Lung cancer is typically characterized by lower survival rates compared to other cancers, and there is a lack of research focused on long-term care specifically for lung cancer. However, in the past several decades, the treatment of lung cancer has become more precise and effective, leading to a decrease in mortality rate and improved survival for patients with non-small cell lung cancer (NSCLC)^4,5^. Survivors of NSCLC are at an increased risk of developing functional disabilities, which can further aggravate the existing risks associated with aging and poor health^6^. Therefore, it is crucial to quantify the extent of functional disability over a lifetime for these patients under long-term care for cancer.

In the modern era of “pay by value”^7–9^, healthcare providers are required to deliver high-quality care which is cost-effective. In general, there are two outcome indicators that are commonly used to measure the value of a disease: one is quality-adjusted life year (QALY), while the other is disability-adjusted life year (DALY)^10^. The former allows for the consideration of both the quantity and quality-of-life gained from different healthcare interventions^9^, while the latter is the sum of the years of life lost (YLL) due to premature death and years living with disability (YLD) with a consideration of the societal impact due to functional disability^11,12^, although the disability weight (DW) of YLD could be influenced by different methodological approaches^13^.

To the best of our knowledge, studies concomitantly measuring the duration or life years of functional disabilities and quality-of-life from a lifetime horizon have been relatively limited, and the comparison of the impacts of health interventions across the whole cycle of prevention, diagnosis, treatment, and care has remained a big challenge for researchers. However, it would be a necessary step to reengineer these interventions and come up with the most cost-effective pathways to improve the sustainability of universal coverage healthcare systems^14^.

Using non-small cell lung cancer (NSCLC) as an example, this study aimed to estimate the long-term impacts of lung cancer on functional disability through integrating a lifetime survival function with the Barthel index (BI) and/or EQ-5D to quantify disability-free life expectancy (DFLE) and QALE. Moreover, we compared them with those of age-, sex-, and calendar year-matched referents to obtain the loss-of-QALE and loss-of-DFLE as alternative indicators for evaluating the potential long-term care needs among survivors of lung cancer.

## Materials and methods

The International Review Board of National Cheng Kung University Hospital approved this study (A-ER-100-079) before commencement. The requirement for informed consent from the study subjects was waived by the International Review Board of National Cheng Kung University Hospital due to the retrospective study design. Study methods were performed in accordance with the STROBE guidelines.

### Data source for lifetime survival

In this study, the databases of the Taiwan Cancer Registry (TCR) and Taiwan National Mortality Registry, provided by the Health and Welfare Data Science Center (HWDC) of the Ministry of Health and Social Welfare, were interlinked to establish the survival function of lung cancer patients. After the interlinkages, all personal identification information was encrypted at the HWDC before data analysis to ensure confidentiality. We abstracted the de-identified demographic and clinical information of patients with non-small cell lung cancer, including sex, age, baseline performance according to the Eastern Cooperative Oncology Group (ECOG) classification, date of diagnosis, survival status, histological subtype, and smoking status. To fit into lung cancer screening policy, we only included NSCLC aged from 50 to 89 years old. All cases were classified according to the American Joint Committee on Cancer (AJCC) system, seventh edition, Ref.^15^ and pathological staging were prioritized to clinical staging. We also obtained national life tables from the Ministry of the Interior of Taiwan to simulate survival functions of the reference population for comparison.

### Hospital cohort for ADL and QOL

All NSCLC patients (the index group) over age 20 who visited National Cheng Kung University Hospital (NCKUH), a national medical center, were invited to fill out self-reported questionnaires, including the EQ-5D and the activities of daily living (ADLs), at each clinical visit from May 2011 to December 2020. Beginning in 2017, patients admitted to the thoracic ward and outpatient clinic of other departments were also included. The scores of each item were abstracted together with each patient’s demographic and clinical data (sex, age, performance status, date of diagnosis, staging, etc.). This study selected only patients with non-small cell lung cancer and stratified them by age (50–64 versus 65–89) and stage (I to IIIa versus IIIb to IV).

### Measurements of functional disability and health utility

The Barthel index (BI) was applied to measure each patient’s functional disability, which was administered by a well-trained and standardized interviewer, or a case manager with a nursing background. The Barthel index is composed of ten items. Each item is rated in terms of whether the patient can perform the self-care tasks or needs assistance based on observation. There are two items (bathing and grooming) assessed with a two-point scale (0 and 5 points), six items (climbing stairs, eating, use of toilet, dressing, bowel control and bladder control) using a three-point scale (0, 5 and 10 points), and two items (moving from a wheelchair to bed and walking on a level surface) using a four-point scale (0, 5, 10, and 15 points). The total score ranges from 0 to 100, with a higher total score indicating better independence. To compare the results with the proportion of people with functional disability in the general population, this study categorized a BI score > 70 as disability-free, which is based on the standard established by a national research project on estimating demand for long-term care services issued by the Council for Economic Planning and Development, Executive Yuan in Taiwan^16^.

The European Quality of Life Five-Dimension (EQ-5D) three-level questionnaire was applied to estimate the utility values of quality of life, in which each patient was invited to fill out the form with partial assistance from our interviewers if needed. The EQ-5D contains five dimensions: mobility, self-care, usual activities, pain or discomfort, and anxiety or depression^17^. By using the scoring function from a Taiwanese study^18^, the results were transformed into utility values ranging from 0 to 1, where 0 represents the worst state and 1 represents the best health condition.

### Statistical analysis

Briefly, we estimated the lifetime survival function to obtain life expectancy (LE), which was multiplied with the QOL utility to calculate QALE. Then, they were compared with the age-, sex-, and calendar year-matched referents simulated from vital statistics to obtain the loss-of-LE and loss-of-QALE. As the Taiwan government adopts an ADL score of equal to or less than 70 as functionally disabled, this study also adopted the same criteria to estimate disability-free LE (DFLE) by multiplying the proportion of people without disability with survival and then summed up to lifetime, while the loss of DFLE was calculated from comparisons with the corresponding referents, detailed as follows.

#### Estimation of life expectancy (LE) and loss-of-LE

We verified the survival status of all NSCLC patients by interlinkage between the Taiwan Cancer Registry and Taiwan National Mortality Registry from 2011 to 2018. The Kaplan–Meier method was initially employed to estimate the survival function, denoted as $S(t|index)$, of the NSCLC population from 2011 to 2018, up to the follow-up limit of the cohort. Subsequently, a semi-parametric extrapolation method^19^ was applied to project the lifetime survival function of the population. The overall extrapolation process involved three main steps: First, we established an age (at-diagnosis)-, sex-, and calendar-year matched referents that corresponded to the index cohort simulated from vital statistics and also estimated by Kaplan–Meier method, denoted as $S(t|ref)$. Second, we constructed survival ratios between the index cohort and the reference population, denoted as $W\left(t\right)=S\left(t|index\right)/S\left(t|ref\right)$. Finally, the survival ratio $W(t)$ was then logit-transformed as $logit\left[W\left(t\right)\right]$ and the data was fitted into a restricted cubic spline model. The goal of this conversion was to align the relative survival in a way that facilitates extrapolation using the matched referents as a splint^19,20^. The extrapolation was then repeatedly conducted using a rolling algorithm, progressing in a month-by-month manner until the entire lifespan of the index cohort reached, or, survival rate < 0.01. The extrapolation method described above was initially proposed, validated, and refined by Hwang and Wang et al., as demonstrated through their studies^19,21,22^. To validate the extrapolation method on lung cancer subcohorts, we have once employed a half-period method in a previous study. We classified the subcohorts based on different pathological types of lung cancer and selected those diagnosed in the first 8 years during the 16-year of follow-up. We then applied our extrapolation method to estimate the outcomes for the full 16-year period. These estimates were compared with the actual Kaplan–Meier estimates of each subcohort up to the end of the 16th year, which served as the gold standard and calculated the relative biases, which ranged below 1.1–7.1%^23^. The method has been applied to different types of cancers^24–26^, end-stage renal disease^27^, acute myocardial infarction^28^, stroke^29^, spinal cord injuries^30^ and rheumatoid arthritis^31^.

The loss-of-life expectancy (loss-of-LE) was calculated by subtracting the life expectancy (LE) of the index population from the life expectancy of the referents, matched by age, sex, and calendar year. All of the aforementioned estimations were performed using the iSQoL2 package in the R software. The iSQoL2 package, developed by Hwang's team for the specific method utilized in this study, is available for free download from the website [http://sites.stat.sinica.edu.tw/isqol/].

#### Estimation of dynamic changes in utility values and proportions of disability-free patients

We adopted the EQ-5D measurements from the 2013 National Health Interview Survey in Taiwan as the QOL utility values for the age-, sex-matched referents. Both the EQ-5D measurements and Barthel index measurements were transformed into values ranging from 0 to 1. By using a Taiwan scoring model for the EQ-5D, we summed the total score of the 5 dimensions and converted it into a utility value, ranging from 0 to 1^18^. Because the utility values for each subgroup were repeatedly measured, we constructed a linear mixed model considering the random effect within each subject. For the BI total score, we defined a value larger than 70 as disability free or 1, and equal to 70 or less than 70 as living with disability or 0. We estimated the kernel smoothing mean by averaging on nearby 10% of sample points of EQ-5D utility value, proportion of people with functional disability, and individual BI score for each subgroup. For instance, if the item ‘moving from a wheelchair to bed’ was measured as 15 points, the patient was considered as free of disability for this item and the value was converted into 1. Whereas if this item was scored as 10, 5 or 0, it would be converted into 0, indicating living with disability.

#### Estimation of disability-free life expectancy (DFLE), loss of DFLE, quality-adjusted life expectancy (QALE) and loss of QALE

In brief, we adopt the general population mortality (obtained from lifetables published in Taiwan vital statistics) to simulate age-, sex-, and calendar year-match referents as a splint for extrapolation^20^. In contrast to Sullivan’s method^32^ mostly rely on period life table, we used cohort lifetables up to the end of follow-up, namely, 2018, and adopt period life table thereafter.

The lifetime survival function was multiplied with the utility value of QOL at each time *t* after diagnosis and summed up throughout life to obtain the QALE. Similarly, the survival function was multiplied or adjusted by the proportion of disability-free patients at time *t* after diagnosis and summed throughout life to obtain the DFLE. Let us denote the survival function of patients with a specific illness as $\widehat{S}\left(t\right)$, while the mean QOL of a patient surviving that illness at time *t* is $Q(t)$ and $\widehat{{P}_{health}}\left(t\right)$ as the disability-free proportion at time *t* after diagnosis. The QALE and DFLE can then be estimated by the following formula:

1 $${\text{QALE}}={\int }_{0}^{\infty }\widehat{S}\left(t\right)\times Q(t)\hspace{0.33em}dt,$$

2 $$\mathrm{DFLE}={\int }_{0}^{\infty }\widehat{\mathrm{S}}\left(\mathrm{t}\right)\times \widehat{{P}_{health}}\left(t\right)dt.$$

Then, we calculated the loss-of-QALE and loss-of-DFLE of each subgroups by subtracting the QALE and DFLE, respectively, of the each index subcohorts form those of the corresponding age-, sex-, and calendar year-matched referents. We also calculated the expected number of years living with disability (EYLD) by subtracting DFLE from LE, which has also been proposed and validated as an estimation for long-term care needs in previous studies^27,29^.

### Ethical issues

The study protocol was approved by the International Review Board of National Cheng Kung University Hospital (A-ER-100-079). The requirement for informed consent from the study subjects was waived by the International Review Board of National Cheng Kung University Hospital due to the retrospective study design. Study methods were performed in accordance with the STROBE guidelines.

## Results

During 2011–2020, we collected a total of 1542 lung cancer patients who were assessed at least once for quality of life or ADLs in NCKU hospital. After excluding 12 patients without staging information, 91 with small cell lung cancer, and 479 without simultaneous measurements of EQ-5D and Barthel index, we recruited 960 cases with NSCLC accompanied with 3088 measurements (Fig. S1). Table 1 summarizes the demographic and clinical characteristics of the included patients from the national cohort extracted from the Taiwan Cancer Registry and those who were assessed with QOL measures in NCKUH (the index group). We found that participants included in this study were generally younger compared with the national cohort (Table S1); 84% of them showed a performance state of 0–1 at baseline, in contrast to 59% in the national cohort; the index group also showed a higher proportion of patients in earlier stages of lung cancer (38% versus 31%), a higher proportion of non-smokers and higher levels of education. In general, our NCKUH sample seemed to have lower prevalence rates of cardiovascular diseases, including stroke, coronary artery disease (CAD), heart failure (HF) and chronic obstructive pulmonary disease (COPD) in comparison with the national cohort.

**Table 1:** Characteristics of NSCLC patients from the national cohort and those collected at National Cheng Kung University Hospital (NCKUH).

	National cohort	NCKUH patients with complete ADL measurements	NCKUH patients without complete ADL measurements
Calendar period (year)	2011–2018	2011–2020	
No. of patients	71,419	960	479
Female, *n* (%)	30,404 (42.6)	451 (47.0)	225(47.0)
Age, *n* (%)			
50–64	26,595 (37.2)	596 (62.1)	249 (52.0)
65–89	44,824 (62.8)	364 (37.9)	230 (48.0)
NSCLC, *n* (%)			
I–IIIA	22,109 (31.0)	365 (38.0)	156 (32.6)
IIIB–IV	49,310 (69.0)	595 (62.0)	323 (67.4)
Performance status, *n* (%)			
0–1	41,976 (58.8)	804 (83.8)	397 (82.9)
2–4	13,401 (18.8)	46 (4.8)	40 (8.4)
Missing	16,042 (22.5)	110 (11.5)	42 (8.8)
Smoking, *n* (%)			
Non smokers	36,385 (51.0)	649 (67.6)	302 (63.1)
Smokers	28,353 (39.7)	311 (32.4)	177 (37.0)
Missing	6681 (9.4)		
Education			
< 12 years	–	500 (52.1)	273 (57.0)
≥ 12 years	–	442 (46.0)	164 (34.2)
Missing	–	18 (1.9)	42 (8.8)
No. employed (%)		271 (30.5)	99 (23.1)
Marital status			
Married	–	713 (74.3)	322 (67.2)
Unmarried/divorced/widowed	–	229 (23.9)	116 (24.2)
Missing	–	18 (1.9)	41 (8.6)
Comorbidity			
Diabetes mellitus	15,436 (21.6)	89 (9.3)	27 (5.6)
Stroke	7029 (9.8)	45 (4.7)	36 (7.5)
Coronary artery disease	8029 (11.2)	19 (2.0)	11 (2.3)
Heart failure	3226 (4.5)	10 (1.0)	8 (2.0)
COPD	10,257 (14.4)	74 (7.7)	57 (12.0)
Liver cirrhosis	1553 (2.2)	21 (2.2)	4 (0.8)
End-stage renal disease	2614 (3.7)	54 (5.6)	26 (5.4)

*NSCLC* non-small cell lung cancer, *ADL* activities of daily living, *COPD* chronic obstructive pulmonary disease.

### QOL utility values and ADL scores

**Figure 1:**
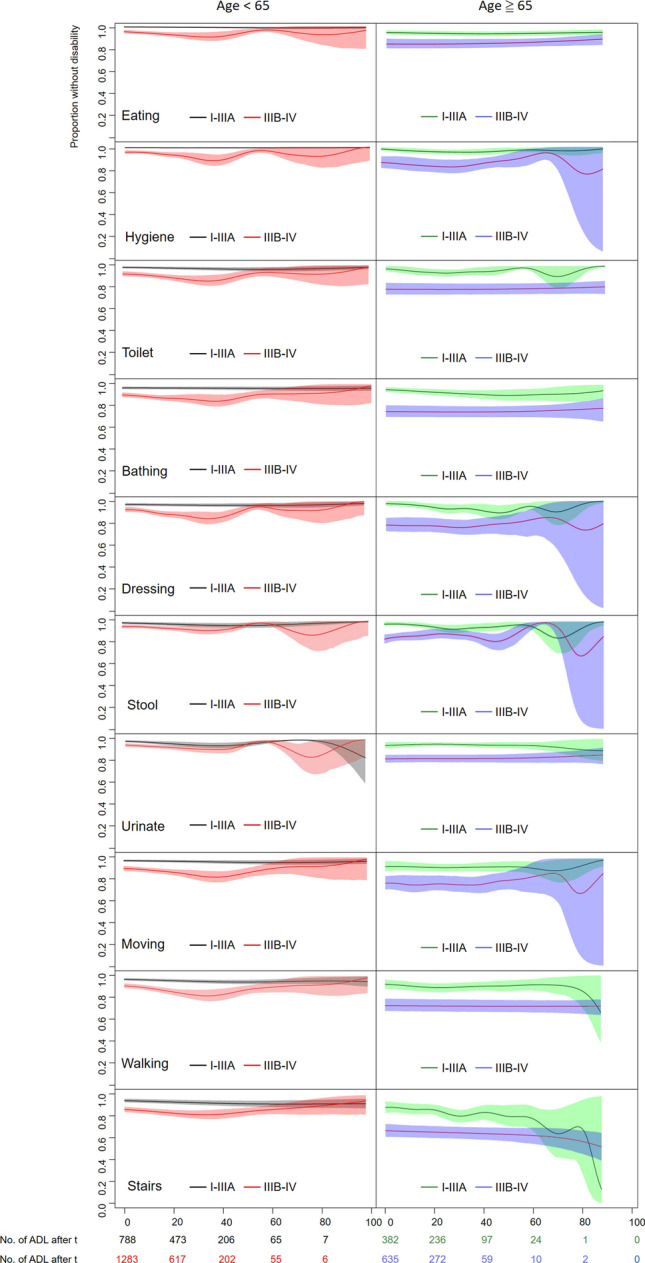
Proportion without disability and 95% confidence limits (colored shadows) for each item of ADL (activities of daily living) by kernel smoothing mean.

Table 2 summarizes the QOL utility values, means of each item and total scores of the Barthel index stratified by age and stage. We found that subjects who were older or diagnosed at an advanced stage of NSCLC showed poorer mean QOL utility values and lower item means and summed ADL scores; the effects associated with advanced (IIIb–IV) stage appeared to be stronger for those aged ≥ 65, as indicated in Table 2 where all the mean utility values, individual and sum scores of those aged < 65 with stage IIIb–IV were consistently lower than those aged ≥ 65 with stage I–IIIa. Figure 1 illustrates the dynamic changes in kernel smoothing mean (and 95% confidence limits highlighted in color) for the proportion of patients without disability for each item at each time *t* after diagnosis; the longer the duration after diagnosis, the lower the proportion of patients with functional disability and the wider the confidence limits for each item due to the smaller sample sizes. Compared with NSCLC patients at the early stage, those at the advanced stage suffered from poorer performance for almost all items except ‘stool’ and ‘urinate’ at the time of diagnosis, which seemed to persist later on. This could be clearly delineated with a larger gap between the two curves, depicting the proportions of patients without disability at early versus advanced stages at the initial diagnosis (Fig. 1), which is usually followed by the overlapping of confidence limits between the curves at 4–5 years after diagnosis.

**Table 2:** Average scores of activities of daily living (ADL) of NSCLC patients collected at NCKUH stratified by age, stage and disability score.

Age	50–64				65–89			
Stage	I–IIIA		IIIB–IV		I–IIIA		IIIB–IV	
ADL sum score	> 70	≤ 70	> 70	≤ 70	> 70	≤ 70	> 70	≤ 70
No. of cases (no. of measurements)	228 (788)		368 (1283)		137 (382)		227 (635)	
No. of cases (no. of measurements)	217 (767)	11 (21)	318 (1146)	50 (137)	130 (362)	7 (20)	182 (497)	45 (138)
Proportions in each group (%)	95.2 (97.3)	4.8 (2.7)	86.4 (89.3)	13.6 (10.7)	94.9 (94.8)	5.1 (5.2)	80.2 (78.3)	19.8 (21.7)
ADL sum score mean (SE)	99.2 (0.11)	53.1 (3.97)	98.8 (0.12)	35.7 (2.00)	98.1 (0.25)	40.3 (5.47)	97.3 (0.25)	31.3 (2.06)
QOL utility value (SE)*	0.90 (0.01)		0.82(0.01)		0.87(0.01)		0.77(0.01)	
Proportion of measurements without disability*	0.97 (0.01)		0.89 (0.01)		0.94 (0.02)		0.80 (0.02)	
No. of measurements without disability (%)	767 (97.3)		1146 (89.3)		362 (94.8)		497 (78.3)	
ADL total score, mean (SE)*	97.9 (0.4)		91.2 (0.9)		94.4 (1.1)		84.3 (1.6)	
Moving, mean (SE)*	14.7 (0.1)		13.6 (0.1)		14.3 (0.2)		12.8 (0.3)	
Walking, mean (SE)*	14.7 (0.1)		13.7 (0.1)		14.2 (0.2)		12.7 (0.3)	
Stairs, mean (SE)*	9.5 (0.1)		8.6 (0.1)		8.6 (0.2)		7.3 (0.2)	
Eating, mean (SE)*	9.9 (0.0)		9.4 (0.1)		9.7 (0.1)		9.0 (0.1)	
Hygiene, mean (SE)*	5.0 (0.0)		4.6 (0.0)		4.8 (0.1)		4.3 (0.1)	
Toilet, mean (SE)*	9.8 (0.0)		9.1 (0.1)		9.6 (0.1)		8.4 (0.2)	
Bathing, mean (SE)*	4.8 (0.0)		4.3 (0.1)		4.6 (0.1)		3.8 (0.1)	
Dressing, mean (SE)*	9.8 (0.0)		9.0 (0.1)		9.6 (0.1)		8.4 (0.2)	
Stool, mean (SE)*	9.9 (0.0)		9.5 (0.1)		9.7 (0.1)		8.9 (0.1)	
Urinate, mean (SE)*	9.8 (0.0)		9.5 (0.1)		9.7 (0.1)		8.6 (0.1)	

*QOL* quality of life, *SE* standard error of mean, *NSCLC* non-small cell lung cancer, *NCKUH* National Cheng Kung University Hospital.

*The total number of patients and total number of measurements were 960 and 3088, respectively. QOL utility values, the proportion of measurements without disability and mean total ADL scores were the intercepts of the linear mixed model. ADL item scaling ranges: 0 and 5 points for hygiene and bathing; 0, 5, 10 points for eating, toilet use, stairs, dressing, stool and urinate; 0, 5, 10, 15 for moving and walking. A patient is defined as ‘without disability’ if ADL total score > 70.

### Loss of LE, loss of DFLE (disability-free life expectancy), EYLD (expected years living with disability) and loss of QALE (quality-adjusted life expectancy)

**Figure 2:**
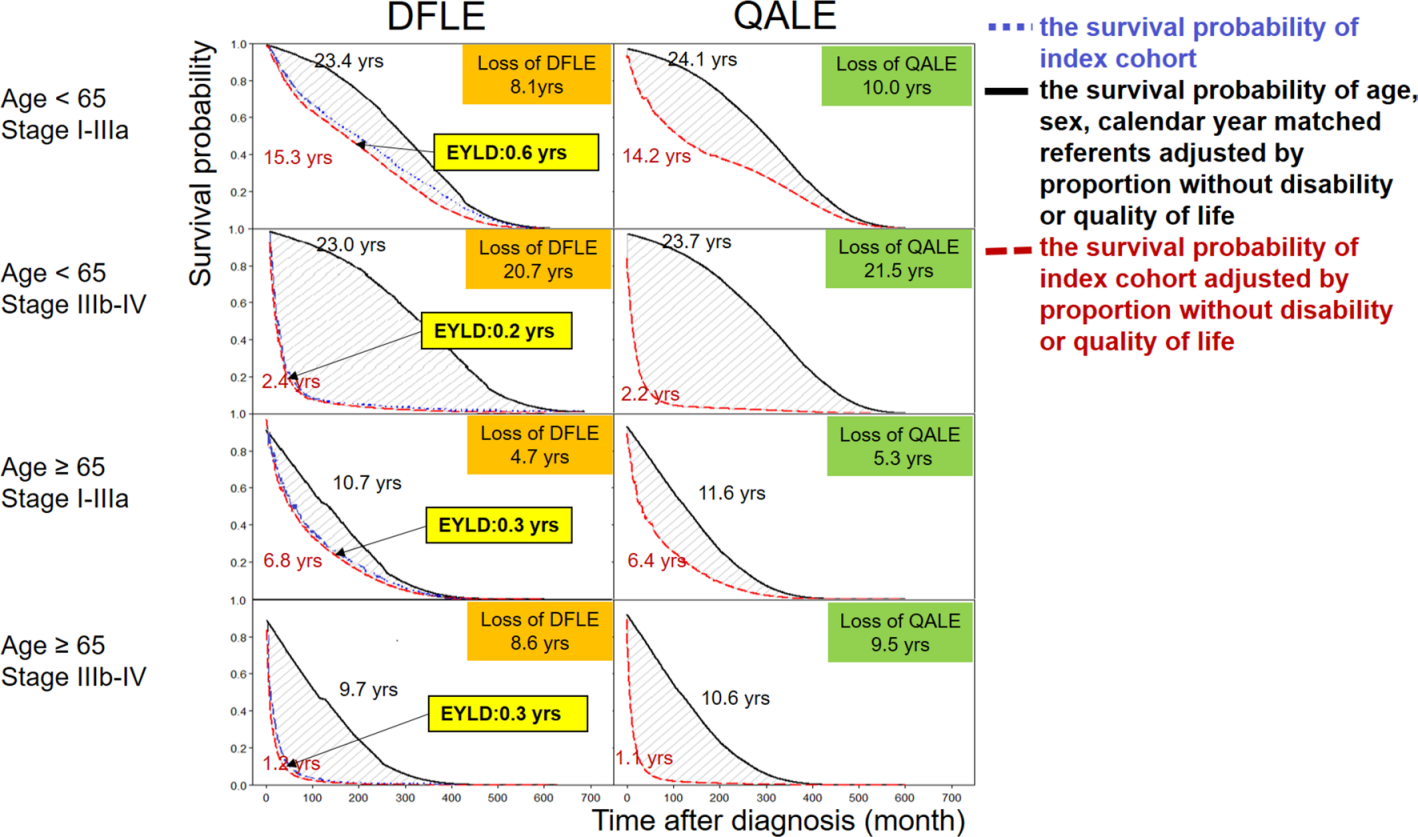
Comparison of disability-free life expectancy (DFLE) and quality-adjusted life expectancy (QALE) in patients with NSCLC (Non-small cell lung cancer). Blue dotted line indicates the survival probability of every index sub-cohort stratified by age and stage. Black line indicates the survival probability of age-, sex-, calendar year-matched referents adjusted by the proportion without disability (left panels) or quality of life (right panels). DFLE is the area under the disability-adjusted survival curve, i.e., survival probability multiplied by the proportion without disability (red dashed lines in left panels), while QALE is the area under the quality-adjusted survival curve, i.e., survival probability multiplied by the utility value of quality of life (red dashed lines in right panels). The losses of DFLE and QALE were calculated by subtracting those of the index sub-cohort from the corresponding referents, as indicated in the shadowed areas. The area between the blue dotted line and red dashed line is the expected years living with disability (EYLD). All numbers are quantified in years (years).

Table 3 summarizes the estimation of LE, loss of LE, DFLE, loss of DFLE, QALE and loss of QALE in each subgroup stratified by age and staging. Figure 2 provides an illustration for comparing the QALE and DFLE among NSCLC sub-cohorts and their corresponding matched referents. In both age groups, we found a consistent trend of decreased LE, QALE, DFLE when the disease progressed to the advanced stage, which exhibited increased loss-of-DFLE and loss-of QALE. The DFLE of NSCLC was generally higher than the QALE in all the stratified sub-cohorts because functional disability is usually the major determinant of quality of life. We also found that in all four subgroups, the loss-of-DFLE was slightly lower than the loss-of-QALE, and the EYLD seemed to be 0.3 to 0.6 years for NSCLC patients (Fig. 2).

**Table 3:** Estimation of life expectancy (LE), disability-free LE (DFLE), quality-adjusted LE (QALE) and respective losses after comparison with age-, sex-, and calendar year-matched referents simulated from vital statistics for patients with NSCLC, stratified by age and stage.

Age and staging	Average age at diagnosis (SD)	Censor rate (%)	LE, years (95% CI)			DFLE, years (95% CI)			QALE, years (95% CI)		
			NSCLC	Ref.	Loss-of-LE	NSCLC	Ref.	Loss-of-DFLE	NSCLC	Ref.	Loss-of-QALE
50–64											
I–IIIA	54.7 (7.5)	81.3	15.9 (13.4–19.3)	26.1 (25.9–26.1)	10.2 (6.8–12.6)	15.3 (13.1–18.7)	23.4 (23.3–23.5)	8.1 (4.7–10.3)	14.2 (12–17.3)	24.1 (24.1–24.2)	9.9 (6.7–12)
IIIB–IV	53.4 (7.7)	27.6	2.6 (2.4–3)	25.5 (25.3–25.5)	22.9 (22.4–23.1)	2.4 (2.1–2.7)	23 (22.9–23)	20.7 (20.3–20.9)	2.2 (2–2.6)	23.7 (23.6–23.8)	21.5 (21.1–21.7)
65–89											
I–IIIA	71.7 (5.3)	55.3	7.1 (6.4–7.8)	13 (12.9–13)	5.9 (5.1–6.5)	6.8 (6.2–7.4)	10.7 (10.6–10.8)	4 (3.3–4.5)	6.4 (5.7–7)	11.6 (11.5–11.7)	5.3 (4.6–6)
IIIB–IV	73.4 (5.7)	18.1	1.5 (1.4 – 1.6)	11.9 (11.8–11.9)	10.5 (10.3–10.5)	1.2 (1–1.3)	9.7 (9.7–9.7)	8.6 (8.4–8.7)	1.1 (1.1–1.2)	10.6 (10.6–10.7)	9.3 (9.2–9.4)

*SD* standard deviation, *Ref.* age at diagnosis-, sex-, and calendar year-matched reference simulated from life tables of vital statistics, *NSCLC* non-small cell lung cancer, *CI* confidence interval.

## Discussion

In this study, our objective was to estimate the lifetime care burden of NSCLC patients using real-world data. Similar to the estimation of DALYs, our quantification of the loss-of-DFLE could be used in economic evaluations in healthcare interventions and cost-effectiveness analysis. Previous studies have reported that tracheal, bronchus, and lung cancer caused an estimated 45.9 million DALYs globally in 2019, with 2.26 million incident cases, resulting in an average burden of approximately 20.3 years of DALY per person^12^. Our study found similar loss-of-DFLE in the age 50–64 and stage IIIb–IV group of NSCLC patients. However, to our best of knowledge, like many of the previous studies, these estimations were predominantly relied on cross-sectional approach over a specific time period and the most of them were derived from consensus from an expert focus group or a sample of general population living in the community^13^. In recent research, there has been a growing recognition of the importance of cohort follow-up methods rather than period method to achieve more continuous estimations^33^. In line with this trend, our study adopted a longitudinal approach and directly measured data from NSCLC patients to estimate the burden of disability. As a result, our estimates would more accurately reflect the potential demand for long-term care among NSCLC patients. Considering the aging population and advancements in medical technology, the demand for long-term care has been steadily increasing. Therefore, it is crucial to allocate appropriate resources to meet these care demands. For example, in Taiwan, the annual budget for long-term care grew from US$ 154 million in 2016 to US$ 2.14 billion in 2022, which did not include the out-of-pocket payments from patients and their families^34^. Moreover, the above estimation could also be included in counting the potential benefits saved from successful prevention of a disease from a societal perspective^10^.

This study has the following strengths in order to accomplish the above goal: First, we recruited all nation-wide subjects from the Taiwan Cancer Registry and interlinked them with the National Mortality Registry to estimate survival. Since every pathologically validated NSCLC patient can be waived from all copayments and the coverage rate of National health insurance (NHI) has been over 99% since 2004, all diagnosed lung cancer patients would be enrolled into our original cohort. Therefore, our sample for survival estimation would be comprehensive and representative. Second, because the 8 years of follow-up exceeds the LE of all sub-cohorts except the sub-cohort with young age and early stage (Table 3, Fig. 2), of which the censored rate was 81%, the extrapolated period to lifetime would not usually be too long. Moreover, our extrapolation method used the mortality of the general population (namely, the lifetables) as a reference to guide the extrapolation^20–22,35^ and we adopted a rolling-over algorithm after the end of follow-up^19^. These estimations have been shown to be relatively accurate and have been empirically applied in several cancer studies^21,24–26^. Third, we abstracted repeatedly measured real world data on QOL and functional disability from a single medical center. We invited all lung cancer patients who visited NCKUH to be assessed with the EQ5D and Barthel index, which is quasi-random and there were no major differences between the respondents and nonrespondents with regards to sex, age, performance status and comorbidities (Table 1). The estimated kernel smoothing mean after diagnosis could be used to adjust the survival function and summed up throughout life to obtain the QALE and DFLE. The above methods have been validated by simulation^35^ and empirically corroborated in previous studies^26,27,36,37^. Because we estimated the dynamic functions of QOL and Barthel index after diagnosis of NSCLC (Fig. 1), the final QALE and DFLE would be more accurate than simply multiplying the mean QOL value and/or Barthel score with LE. Fourth, all data on the referents were collected from real world databases: the survival functions of the age-, sex-, and calendar year-matched referents were simulated from Taiwan’s national vital statistics; the quality of life functions were taken from the Taiwan National Health Interview Survey in 2013; the age-, sex-specific proportions of disability of the general population were abstracted from the Taiwan national demand estimation for long-term care in 2009^16^; and both QOL and disability functions were individually multiplied with the corresponding survival functions to estimate the QALE and DFLE of the referents. We thus tentatively conclude that our estimates of LE, QALE, DFLE and the corresponding loss-of-LE, loss-of-QALE, and loss-of-DFLE would be relatively accurate and could be applied to health policy decision making (Table 3, Fig. 2). Finally, in comparison to the methodology employed in prior studies for evaluating health-adjusted life expectancy, such as the commonly used Sullivan method^32^, our study also takes into account of specific age-specific mortality rates and disability rates. While these original methods typically rely on period life tables due to limited access to cohort data, our study utilizes cohort life tables, allowing for a longitudinal perspective in estimating DFLE for a specific disease. In addition, the estimation of DFLE for the reference group in our study involves utilizing the survival function derived from national life tables, which is then multiplied by age-specific disability rates obtained from a nationwide long-term care database. This comprehensive approach enables us to capture the disability-adjusted life expectancy of the general population at a national level. Consequently, by subtracting the calculated DFLE for the NSCLC subcohorts, the resulting loss-of-DFLE provides a more precise measure of the disease-specific impact on the social burden. In contrast to the conventional method of using multistate lifetables^38^ with many assumptions and parameters to be estimated, we provide a simplified, alternative approach to directly employ real world data (namely, Barthel index) for estimation of health effects resulted from different technology on a specific illness.

Several implications for policy making could be inferred from this study: First, in a typical cancer cohort, we anticipate both older age and advanced stages would result in lower DFLE and QALE compared with younger age and earlier stages. This study, however, showed that advanced stage had a larger impact than older age in patients with NSCLC for individual items and summed scores as well as utility (Table 2, Fig. 2). Moreover, Fig. 1 indicates bigger gaps for most items of ADL between early and advanced stages right from after diagnosis and usually lasted for more than 5 years, while the gaps between young and old ages seemed less obvious. We speculate that this might have resulted from deteriorated pulmonary function due to lung collapse or pleural effusion^39^ in advanced-stage patients rather than due to aging^40^. Second, since one of the 5 dimensions in the EQ-5D is self-care, which is measured in more detail in the Barthel index, we found that the DFLE in each sub-cohort was almost the same or very close to the QALE. It corroborates the hypothesis that functional disability would usually be the major determinant of quality of life^41^, including for NSCLC patients (Table 3). Third, the number of years living with disability estimated from real-world data appeared to be small due to the short life expectancy of NSCLC (Fig. 2). The results are compatible with the general assumption that loss-of-LE, loss-of-QALE, and loss-of DFLE would be higher in younger age groups since they would have a longer life expectancy (Table 3). Overall, these findings provide valuable insights for healthcare policymakers in terms of resource allocation and the development of comprehensive care pathways that integrate prevention, diagnosis, treatment and long-term support for cancer survivors. The study contributes to the evaluation of strategies aimed at improving the sustainability of healthcare institutions and reducing the burden on families caring for non-autonomous individuals^14^.

### Limitations

There are several limitations in this study that must be acknowledged. First, the reference group for disability was based on information from about a decade ago^16^.

This may lead to an underestimation of DFLE and care burden in the reference group. Therefore, we must update the prevalence of disability in the general population regularly to develop fair and effective policies for resource allocation. Second, although we tried to consider as many comorbidities as possible to account for possible functional disabilities, the measurements were usually more comprehensive in the NCKUH sample than those recorded in the NHI database. In the NCKUH medical center, we were able to abstract all the diagnoses from medical records, however, the NHI currently allows only 3 major diagnoses for data recorded in clinics and 5 diagnoses for data collected in hospitals for reimbursement. Nevertheless, the possibilities of other factors that would affect functional disability such as blindness or amputations are usually low, thus the results would not be biased too much. Third, as patients with NSCLC usually die within 5 years, the number of patients still eligible for ADL measurements usually decreases over time and the confidence limits would become wider. However, as our estimation was adjusted by the survival rate at each time point, after multiplying the survival function by the disability proportion, the potential bias of life expectancy would be negligible. Fourth, since there were no direct ADL measurement data in our national database, these data were all collected from NCKUH and we integrated them with national survival data to calculate the DFLE. The representativeness of such a hospital cohort would be a concern as all our patients with lung cancer were recruited mostly from outpatient clinics, so they were generally younger, had better performance status at baseline, and were less comorbid with cardiovascular disease (Table 1). Because NSCLC patients with poorer ECOG performance have higher odds of moderate to severe mobility disability compared to those with no or slight disability^42^, this study could have underestimated the overall impact of the disease, namely, the loss-of-QALE and loss-of-DFLE. Fourth, the usual life expectancy for female lung cancer patients would be longer compared to males^43^. However, due to the smaller sample size available for both quality of life and disability assessments, we did not stratify gender groups in analyzing QALE and DFLE. Therefore, we aimed to focus on differentiating potential effects of age versus advanced stages of lung cancer under limited data. Future studies are warranted to recruit a larger sample sizes, in which one would be able to stratify the above estimations by gender more accurately. Lastly, since extrapolation method employed in this study relied heavily on real-world data, it generally requires a sufficiently long follow-up period to achieve a certain level of accuracy. Typically, this period ranges from 5 to 10 years or more, depending on the specific disease. Therefore, factors such as data accessibility, accuracy, and the duration of follow-up may limit the feasibility of our estimation methods in other countries.

## Conclusion

This study reveals significant decreases in DFLE among survivors of NSCLC, particularly in younger individuals diagnosed at advanced stages. Functional disability emerges as a key determinant of NSCLC patients’ quality of life, impacting both DFLE and QALE. Recognizing the anticipated rise in long-term care demands associated with functional disability is imperative to address this societal burden in future health technology assessment studies. These findings highlight the importance of enhancing cancer prevention policies, treatments, and long-term management, with a specific focus on functional health in NSCLC survivors. Further research across diverse cancer types is recommended to inform comprehensive cancer prevention strategies.

### Supplementary Information

Supplementary Information.

## Data Availability

The datasets generated and/or analyzed during this study are not publicly available due to confidentiality reasons, but the sufficiently aggregated data used for analyses may be available from the corresponding author upon reasonable request.
