# Supplementary material for: Estimation of disability free life expectancy in non small cell lung cancer based on real world data
Source: Sci Rep. 2023 Aug 16;13:13318. doi: 10.1038/s41598-023-40117-5 (PMC10432474; doi:10.1038/s41598-023-40117-5)
Supplement: Supplementary file 1 — Supplementary Information. [file 41598_2023_40117_MOESM1_ESM.docx]

**Supplementary information**

**Figure S1.** Flow diagram of a more detailed sample selection process including ADL (activity daily living), QoL (quality of life) measurements in the NCKU cohort. “N” indicates the number of patients, while “No.” indicates the number of measurements.

**
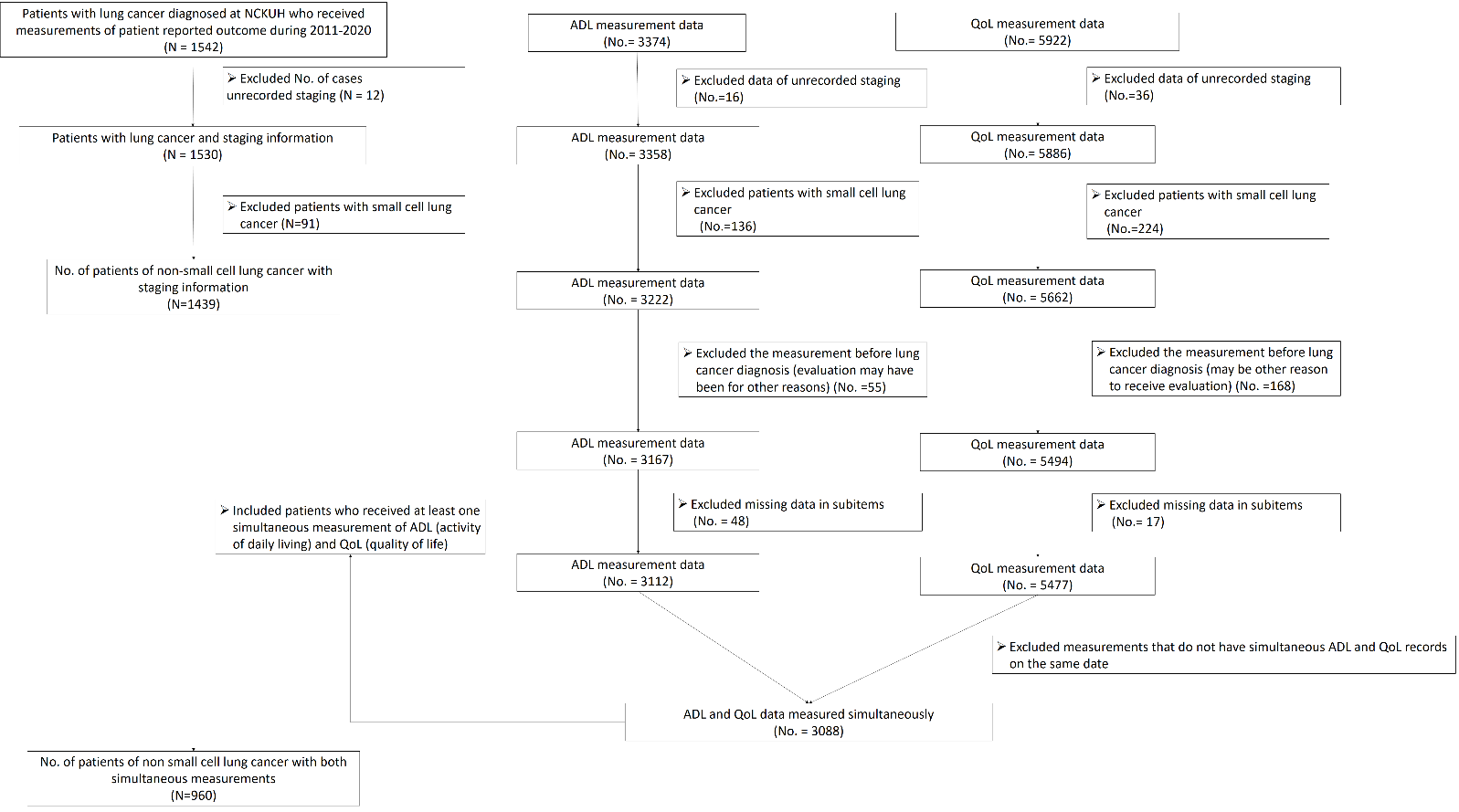
**

**Table S1.** Comparison of characteristics of NSCLC patients from the national cohort and those collected at National Cheng Kung University Hospital (NCKUH) with ADL measurements.

|  | **National cohort** | **NCKU cohort with complete**  **ADL measurements** | **Absolute value of standardized mean difference (SMD)*** |
| --- | --- | --- | --- |
| Calendar year | 2011-2018 | 2011-2020 |  |
| No. of patients | 71419 | 960 |  |
| Sex |  |  | 0.09 |
| Male, n(%) | 41015 (57.43) | 509 (53.0) |  |
| Female, n (%) | 30404 (42.57) | 451 (47.0) |  |
| Age, *n (%)* |  |  | 0.51* |
| 50-64 | 26595 (37.24) | 596 (62.1) |  |
| 65-89 | 44824 (62.76) | 364 (37.9) |  |
| Stages, *n (%)* |  |  | 0.15 |
| I-IIIA | 22109 (30.96) | 365 (38.0) |  |
| IIIB-IV | 49310 (69.04) | 595 (62.0) |  |
| Performance status, *n (%)* |  |  | 0.58* |
| 0-1 | 41976 (58.77) | 804 (83.8) |  |
| 2-4 | 13401 (18.76) | 46 (4.8) |  |
| Missing | 16042 (22.46) | 110 (11.5) |  |
| Smoking, *n (%)* |  |  | 0.34* |
| Non smokers | 36385 (50.95) | 649 (67.6) |  |
| Smokers | 28353 (39.70) | 311 (32.4) |  |
| Missing | 6681 (9.35) |  |  |
| **Comorbidity** |  |  |  |
| Diabetes mellitus | 15436(21.6) | 89 (9.3) | 0.35 |
| Stroke | 7029(9.8) | 45 (4.7) | 0.20 |
| Coronary artery disease | 8029(11.2) | 19 (2.0) | 0.38 |
| Heart failure | 3226(4.5) | 10 (1.0) | 0.22 |
| COPD | 10257(14.4) | 74 (7.7) | 0.21 |
| Liver cirrhosis | 1553(2.2) | 21 (2.2) | 0.00 |
| End-stage renal disease | 2614(3.7) | 54 (5.6) | 0.09 |
| NSCLC: Non-small cell lung cancer; ADL: Activities of daily living  *An absolute value of less than 0.2 suggests that the two groups are balanced. | | | |
